# Nikolavsky Urethroplasty for Distal Urethral Stricture in a Young Man With Severe Obesity and Lichen Sclerosus: A Surgical Approach to a Rare Presentation

**DOI:** 10.7759/cureus.95521

**Published:** 2025-10-27

**Authors:** Joseph McGrath, Alexander Blanca, Ashley Gordon, Laura Horodyski

**Affiliations:** 1 Medicine, Nova Southeastern University Dr. Kiran C. Patel College of Osteopathic Medicine, Davie, USA; 2 Urology, University of Miami Leonard M. Miller School of Medicine, Miami, USA

**Keywords:** bmg urethroplasty, distal urethroplasty, lichen sclerosus, penile lichen sclerosus, reconstructive urology, recurrent urinary tract infection, severe obesity, transurethral ventral buccal mucosa graft, urethral stricture, urology

## Abstract

This case report describes a rare occurrence of distal urethral stricture secondary to lichen sclerosus (LS) in a 19-year-old man, an inflammatory condition typically seen in older individuals. The patient had a medical history notable for recurrent urinary tract infections (rUTIs), phimosis, nephrolithiasis, and class III obesity (BMI 53.4). He presented with dysuria and incomplete bladder emptying, which mildly improved with tamsulosin. Physical examination revealed pinpoint meatal stenosis. Cystoscopy with retrograde urethrography demonstrated a 2.5-cm distal penile urethral stricture with a normal-appearing posterior urethra. A transurethral ventral buccal mucosa graft inlay urethroplasty (Nikolavsky urethroplasty) was performed as a single-stage operation. Postoperatively, the patient recovered well without complications and demonstrated marked improvement in urinary symptoms and resolution of rUTIs during follow-up, with no need for additional interventions. The patient’s history of rUTIs likely accelerated stricture progression by worsening the inflammatory response associated with LS. This case highlights the potential contribution of severe obesity as a risk factor for LS and urethral strictures and underscores the importance of considering LS as a possible etiology in younger patients. It also supports the efficacy of buccal mucosa grafts as a durable and effective approach for complex urethral reconstruction.

## Introduction

Lichen sclerosus (LS) is a chronic inflammatory condition that predominantly affects the anogenital region and is associated with urethral strictures in males. It occurs approximately 6-10 times more frequently in women than in men, with peak prevalence in males typically in the sixth decade of life. LS, previously known as balanitis xerotica obliterans (BXO), was first identified in the nineteenth century, with the male form described by Stuhmer in 1928. It is now recognized as one of the most common causes of long-segment urethral strictures in men, affecting the urethral epithelium and leading to progressive narrowing and dysfunction of the urethra [1-3]. Although obesity has been hypothesized to contribute to the pathogenesis of LS through mechanisms involving chronic inflammation and poor genital hygiene, data directly examining this association remain limited. One of the few studies investigating this relationship found that men with LS-related urethral strictures had significantly higher body mass indices compared to those with non-LS strictures [4].

When LS involves the distal penile urethra, surgical intervention is often required to restore urinary function and prevent complications such as acute urinary retention, recurrent urinary tract infections (rUTIs), and renal impairment. The transurethral ventral buccal mucosa graft inlay urethroplasty (Nikolavsky urethroplasty), a technique first described within the last decade by Dr. Nikolavsky, offers a single-stage approach for such cases. Buccal mucosa grafts are considered ideal for urethral reconstruction due to their durability, mucosal compatibility, and resistance to fibrosis, demonstrating high success rates and a favorable safety profile, particularly in LS-related anterior urethral strictures [5-7].

Urethral stricture disease itself is relatively uncommon, with an estimated prevalence of 0.6% in the general male population. However, prevalence increases to 1%-2% among men aged 55 and older, while in men under 45 years old, it remains around 0.3% [8]. This report describes a rare case of LS in a severely obese 19-year-old man with rUTIs, presenting with an anterior urethral stricture extending 2.5 cm proximally from the meatus.

## Case presentation

A 19-year-old man was admitted to the hospital for a urinary tract infection (UTI). His past medical history was significant for rUTIs, nephrolithiasis, phimosis, and LS of the glans penis. The patient reported dysuria and incomplete bladder emptying. He denied any family history of urological or other malignancies. Social history was negative for alcohol use, substance abuse, and cigarette or electronic vaping use. His body mass index (BMI) was 53.4, consistent with class III obesity.

Past surgical history included circumcision during infancy. The patient had experienced two prior UTIs earlier in the year, both empirically treated with trimethoprim-sulfamethoxazole. Although the urine culture for the most recent infection was negative, a previous culture obtained one month earlier had grown *Klebsiella pneumoniae*.

**Figure 1:**
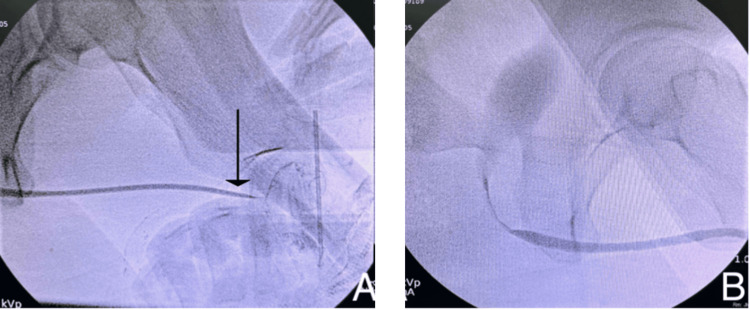
Preoperative retrograde urethrography demonstrating a 2.5 cm distal penile urethral stricture (A; black arrow) and a normal-appearing posterior urethra (B)

Cystoscopy with retrograde urethrography (RUG) demonstrated a distal penile urethral stricture measuring 2.5 cm in length, extending proximally from the meatus, with a normal-appearing posterior urethra (Figure 1).

Findings from the RUG were consistent with LS causing a distal penile urethral stricture. An 8 Fr ureteroscope could not be advanced through the stricture. Given the patient’s young age, as well as the location, length, and LS involvement, the treatment team and patient elected to proceed with a transurethral ventral buccal mucosa graft inlay urethroplasty (Nikolavsky urethroplasty). Tamsulosin provided mild symptomatic relief while awaiting surgery.

On the day of the procedure, the patient was brought to the operating room in the supine position and administered gentamicin and cefazolin for prophylaxis. Methylene blue dye was instilled into the urethra to delineate the stricture. A ventral urethrotomy was performed and extended proximally. Flexible cystoscopy revealed LS changes involving the glans penis, a very narrow urethral stricture extending 2.5 cm proximally from the meatus, a normal-appearing posterior urethra, and no bladder lesions.

A transurethral ventral buccal mucosa graft inlay urethroplasty was then performed to reconstruct the anterior urethral stricture, following the technique described by Nikolavsky [5]. A buccal mucosal graft measuring 2.5 × 2.5 cm in a triangular configuration was harvested from the left cheek and secured as a ventral inlay. A silicone catheter was placed and planned to remain in situ for two weeks. No intraoperative complications were encountered.

Postoperative management included daily use of Magic Mouthwash (containing diphenhydramine, aluminum hydroxide, magnesium hydroxide, and 2% lidocaine), a 10-day course of trimethoprim-sulfamethoxazole, and oxybutynin while the Foley catheter remained in place. The patient reported marked improvement in urinary symptoms and resolution of recurrent UTIs during follow-up visits.

## Discussion

Distal urethral strictures in young males, particularly those secondary to LS, are relatively rare but present significant clinical challenges. LS, previously known as BXO, is a chronic inflammatory condition primarily affecting the anogenital region. It leads to progressive fibrosis and atrophy of the genital skin and urethral epithelium, ultimately resulting in urethral stricture formation [1]. Although LS is most commonly diagnosed in older males, particularly in the sixth decade of life, the disease can also manifest at a younger age, as demonstrated in this 19-year-old patient.

The relationship between obesity and LS-related urethral strictures remains unclear; however, emerging evidence suggests a possible association. This patient’s BMI of 53.4 raises important clinical considerations. LS-related urethral strictures have been linked to elevated BMI and comorbid metabolic conditions such as diabetes, hypertension, and hyperlipidemia. These findings support the hypothesis that systemic inflammation and metabolic dysregulation may contribute to disease onset and progression in genital and urethral tissues [4,9]. The precise mechanisms behind this association remain under investigation, highlighting the need to consider metabolic risk factors when evaluating patients with LS-related urethral pathology.

LS is one of the leading causes of long-segment urethral strictures in males, most commonly involving the distal penile urethra and meatus [1,3]. Although the disease can remain asymptomatic for extended periods, rUTIs may exacerbate inflammation and fibrosis, accelerating stricture formation. In this patient, the combined effects of LS, rUTIs, and severe obesity likely contributed to the development of the distal urethral stricture, consistent with other reported cases [10].

The Nikolavsky urethroplasty, or transurethral ventral buccal mucosa graft inlay urethroplasty, has emerged as a novel and effective technique for managing LS-related strictures, especially those beginning at the meatus and extending proximally. Buccal mucosa is an ideal graft material due to its durability, resistance to fibrosis, ease of harvest, and high vascularity, which facilitates rapid graft integration. Its mucosal properties closely resemble those of the urethral lining, making it particularly suitable for urethral reconstruction [5].

In this case, the Nikolavsky urethroplasty provided several advantages. It enabled reconstruction of the narrowed distal urethra in a single-stage procedure, with reported long-term patency rates exceeding 80% [5,11,12]. The technique also reduces the risk of recurrence, urinary retention, recurrent infections, and the need for long-term catheterization [7,12]. Buccal mucosa grafts are especially advantageous in LS-related strictures, as they better resist the progressive scarring typical of the disease compared to skin grafts. This makes them a durable option both for addressing the stricture and for counteracting the fibrotic changes associated with LS. Although LS has traditionally been associated with higher urethroplasty failure rates, early outcomes of the Nikolavsky technique have shown promising success, even in distal strictures caused by LS. While comparative studies remain limited, their results appear favorable relative to conventional approaches, which often show higher recurrence rates in LS-related cases [5,6].

Obesity has also been explored as a potential factor affecting urethroplasty outcomes. Breyer et al. found that overweight (BMI 25-30) and obese (BMI 30-35) patients had a higher risk of recurrence, whereas those with severe obesity (BMI > 35) did not, suggesting a nonlinear relationship between BMI and urethroplasty failure risk [13]. Conversely, Rapp et al. reported no significant association between BMI and recurrence, with multivariate analysis failing to identify BMI as a predictive factor [14]. These conflicting findings underscore the need for further research on the role of obesity in urethroplasty outcomes, as current evidence remains inconclusive and may be influenced by other variables such as stricture complexity and surgical technique.

In summary, this case highlights the effectiveness of the Nikolavsky urethroplasty as a single-stage treatment option for young patients with LS-related distal urethral strictures and underscores the potential role of obesity in the underlying inflammatory process. This approach not only resolves the mechanical obstruction but also helps interrupt the cycle of inflammation and infection, ultimately improving quality of life and reducing the risk of recurrent urological complications.

## Conclusions

This report describes an unusually early presentation of an LS-related distal urethral stricture in a 19-year-old male, a condition typically observed in older adults. The patient’s history of rUTIs likely accelerated the progression of the stricture, underscoring the role of inflammation in LS-related urethral disease, particularly in the context of severe obesity. The Nikolavsky urethroplasty, using a buccal mucosa graft, proved to be an effective single-stage treatment that achieved long-term patency and reduced the risk of recurrence. This case emphasizes the importance of recognizing LS as a potential etiology of urethral strictures in younger patients, the possible influence of obesity on LS progression and surgical outcomes, and the utility of buccal mucosa grafts in the reconstruction of complex anterior urethral strictures.
